# How physicians embrace AI: insights from technology acceptance and trust theories

**DOI:** 10.3389/fdgth.2026.1722087

**Published:** 2026-03-13

**Authors:** Fatma Nur Cicin, Güney Çetin Gürkan

**Affiliations:** 1 Faculty of Economics, Administrative and Social Sciences, Department of Management Information Systems, Istinye University, Istanbul, Türkiye; 2 Faculty of Applied Sciences, Department of Tourism Management, Trakya University, Edirne, Türkiye

**Keywords:** artificial intelligence, healthcare, physicians, technology acceptance, technology acceptance model, theory of planned behavior, trust

## Abstract

**Objective:**

This study investigates the factors influencing physicians’ acceptance and adoption of artificial intelligence (AI) technologies in clinical practice, integrating the Theory of Planned Behavior (TPB) and the Technology Acceptance Model (TAM), while also examining the mediating role of trust.

**Methods:**

A structured survey was conducted among 414 physicians assessing their perceptions of AI technologies using constructs from TPB, TAM, and trust-related factors. Partial Least Squares Structural Equation Modeling (PLS-SEM) was employed for data analysis.

**Results:**

Findings confirm that TPB and TAM effectively explain physicians’ AI acceptance, with TPB exhibiting a stronger predictive power compared to TAM. Trust emerged as a critical determinant in AI adoption, fully mediating the relationship between perceived behavioral control (*p* < 0.001), subjective norms (*p* < 0.05), perceived usefulness (*p* < 0.001), ease of use (*p* < 0.001), and behavioral intention. Notably, perceived ease of use (*p* < 0.001) had the strongest direct impact on trust, while perceived usefulness (*p* < 0.001) significantly influenced behavioral intention. Attitude toward AI showed a significant effect (*p* < 0.01). Subjective norms and perceived behavioral control had weaker direct influences (*p* < 0.05 and *p* = 0.07, respectively).

**Conclusion:**

Trust plays a pivotal role in AI adoption, shaping physicians’ acceptance beyond traditional TPB and TAM factors. Healthcare administrators, policymakers, and technology developers should focus on enhancing trust by improving AI transparency, interpretability, and user-friendly design.

## 1 Introduction

The global healthcare system is undergoing a profound transformation driven by population ageing, the rise of chronic diseases, increasing demand for quality and accessibility, and economic pressures. In response to these challenges, recent advances in artificial intelligence (AI), machine learning (ML), big data, and the Internet of Things (IoT) are reshaping healthcare delivery (1, 2). Despite the growing evidence of AI's transformative potential in medicine, the actual integration of AI tools into clinical practice remains limited. Physicians, as the primary users and gatekeepers of medical technologies, often express hesitation towards AI adoption. This reluctance is attributed to concerns around ethical accountability, explainability, reliability, and data privacy (3).

Previous studies have predominantly focused on the technical performance of AI or general perceptions among healthcare staff, often neglecting the psychological and behavioural mechanisms that underpin physicians' acceptance decisions (4). This gap highlights the need for a theoretically grounded understanding of how and why physicians embrace or resist AI technologies. To address this need, our study draws on two widely validated theoretical frameworks: the Theory of Planned Behavior (TPB) and the Technology Acceptance Model (TAM).

TAM has been extensively applied to explain technology adoption, particularly in clinical and information system settings, due to its emphasis on perceived usefulness and perceived ease of use (5, 6). However, it tends to overlook important social and contextual influences such as subjective norms and perceived behavioural control. In contrast, TPB provides a broader behavioural framework by incorporating attitudes, social pressures, and perceived control over behaviour (6). Nevertheless, TPB does not directly address the usability aspects of technology systems. To overcome these limitations, several studies have suggested integrating TAM and TPB to offer a more comprehensive perspective on technology acceptance (7, 8). In addition, trust has emerged as a critical yet underexplored mediating variable in the adoption of AI systems in healthcare settings, influencing clinicians' willingness to rely on algorithm-based decision support (9).

Although the Unified Theory of Acceptance and Use of Technology (UTAUT) model provides a comprehensive framework for technology adoption (10), this study employed TAM and TPB to maintain comparability with prior physician-focused acceptance studies and to explicitly model trust as a mediating construct.

The aim of this study is to investigate physicians' acceptance of AI technologies in healthcare by integrating the TAM, the TBP, and trust into a unified explanatory framework. Specifically, the study examines how TAM and TPB constructs influence physicians' trust in AI systems and, in turn, how trust affects behavioural intention to adopt AI technologies. By testing this integrated model on a nationwide physician sample, the study seeks to provide empirical evidence on the key psychological and organisational factors shaping AI adoption in clinical practice.

The remainder of this paper is organised as follows. Section 2 presents the theoretical background of TPB, TAM, and trust. Section 3 describes the research model, hypotheses, data collection, and analytical methods. Section 4 reports the results of measurement and structural model analyses. Section 5 discusses the findings, limitations, and future research directions. Finally, Section 6 concludes the study.

## 2 Literature review

### 2.1 Theory of planned behavior (TPB)

TPB, developed by Ajzen (9), is a prominent model in social psychology that explains how individual attitudes, subjective norms, and perceived behavioural control influence behavioural intentions. In healthcare contexts, TPB has been widely applied to understand professionals' decisions regarding technology use, clinical guideline adherence, and adoption of evidence-based practices (11–13).

Attitude toward behaviour refers to an individual's overall evaluation of performing a specific action, ranging from favourable to unfavourable. In healthcare settings, physicians who perceive AI as a means to improve diagnostic accuracy or increase efficiency are more likely to develop a positive attitude, which in turn enhances their intention to adopt such technologies. Subjective norms denote the perceived social pressures that influence an individual's behavioural decisions. For medical professionals, acceptance of AI is often shaped by the expectations of colleagues, organisational practices, and guidance from professional associations. Perceived behavioural control reflects the extent to which individuals feel capable of performing the behaviour in question. In the context of AI adoption, this includes the availability of sufficient knowledge, technical infrastructure, and training opportunities—all of which are essential for the effective integration of AI into clinical workflows.

TPB's strength lies in its ability to account for both individual and contextual factors, making it particularly useful for understanding healthcare professionals' behavioural intentions toward new technologies (14).

### 2.2 Technology acceptance model (TAM)

TAM, introduced by Davis (15), has been extensively used to explain user acceptance of information systems. The model posits that perceived usefulness and perceived ease of use are the primary determinants of an individual's intention to use a technology.

In healthcare, TAM has been applied to study the adoption of electronic health records, decision support systems, and AI-based tools (8, 16, 17). For instance, if physicians believe that an AI system improves diagnostic accuracy and is intuitive and user-friendly, their intention to use it is likely to increase. While TAM offers strong explanatory power in technological contexts, it lacks constructs that capture social influences or perceived control over behaviour, which are critical in clinical environments.

### 2.3 Integrating TPB and TAM with trust

Given their complementary strengths, integrating TAM and TPB allows for a more holistic understanding of physicians’ behavioural intentions by combining technological, personal, and contextual factors (6, 18). However, prior studies have suggested that these frameworks alone may not fully capture the psychological dynamics involved in adopting complex and opaque technologies such as AI (7).

Trust has thus emerged as a crucial mediating factor in technology acceptance models, particularly in healthcare, where perceived risk and uncertainty are high (9, 19) Trust in AI involves confidence in its safety, reliability, transparency, and alignment with professional values. Physicians' trust is shaped not only by the system's performance but also by social and institutional cues—such as peer endorsement, regulatory approval, and explainability features (20, 21).

Empirical studies have shown that trust enhances the impact of perceived usefulness and ease of use on intention to adopt technology, and it may even substitute for attitude when risk sensitivity is high (22, 23). Therefore, this study integrates trust as a mediating construct between TPB/TAM components and behavioural intention, aiming to provide a more comprehensive framework for understanding AI adoption among physicians.

## 3 Material and method

### 3.1 Research model and hypotheses

The dependent variable in the proposed model is physicians' behavioural intention to use AI technologies. Prior research has shown that behavioural intention is a robust predictor of actual system usage in healthcare information systems (10, 17).

Given that AI technologies are still in early stages of adoption in many healthcare settings—and that physicians' exposure to such systems varies widely—this study focuses specifically on intention rather than actual usage (1, 2, 24). This allows us to evaluate the determinants of readiness and acceptance, even among users with limited prior experience.

### 3.2 Research hypotheses

**Figure 1:**
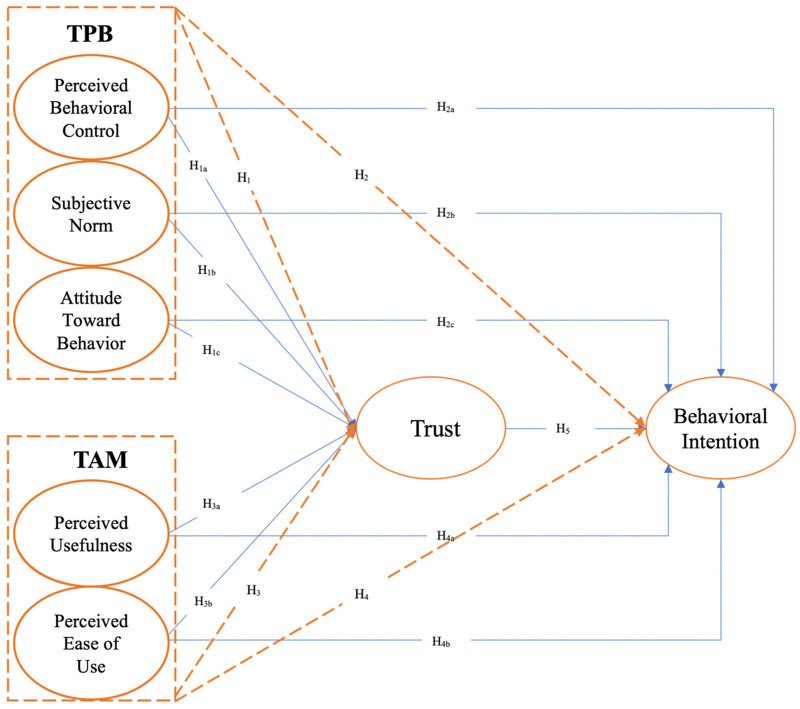
The proposed research model integrates constructs from the theory of planned behavior (TPB) and the technology acceptance model (TAM), emphasizing the mediating role of trust in shaping behavioral intention.

To operationalise the integrated model, a set of hypotheses was developed that reflects both direct and indirect (mediating) effects of key constructs, as illustrated in Figure 1. The hypotheses are grouped according to their functional roles in the model:

#### 3.2.1 Direct effect hypotheses

TBP assumes that attitudes, subjective norms, and perceived behavioural control influence behavioural intentions and related psychological states (11). In the context of AI adoption in healthcare, these constructs are also expected to shape physicians' trust in AI systems. Given the uncertainty, opacity, and perceived risk associated with clinical AI tools, physicians' confidence in their ability to use such systems (perceived behavioural control), perceived professional expectations (subjective norms), and personal evaluations of AI (attitude) are likely to influence trust formation (10, 20).

Empirical evidence supports these associations in medical AI contexts. Fan et al. demonstrated that healthcare professionals' perceptions of system characteristics and social influence significantly affect trust in AI-based diagnostic support systems (20). Similarly, Choudhury et al. reported that expectancy-related beliefs and perceived risk directly shape clinicians' trust in AI-driven clinical decision tools (9). These findings suggest that TPB-related beliefs play a critical role in establishing trust toward AI technologies in healthcare settings.

Accordingly, the following hypotheses are proposed:

- **H1:** TPB has a statistically significant effect on trust.
  - **H1a:** Perceived behavioral control has a statistically significant effect on trust.
  - **H1b:** Subjective norms have a statistically significant effect on trust.
  - **H1c:** Attitude has a statistically significant effect on trust.

TPB has been widely validated in predicting technology acceptance through its three antecedents (11). In the context of AI adoption, physicians' beliefs about its benefits (attitude), social endorsement (subjective norms), and perceived ease of engaging with AI systems (perceived behavioural control) are expected to directly influence their behavioural intention (20, 25);

- **H2:** TPB has a statistically significant effect on behavioral intention.
  - **H2a:** Perceived behavioral control has a statistically significant effect on behavioral intention.
  - **H2b:** Subjective norms have a statistically significant effect on behavioral intention.
  - **H2c:** Attitude has a statistically significant effect on behavioral intention.

TAM suggests that system-related beliefs such as perceived usefulness and perceived ease of use shape user attitudes toward technology (14). Beyond attitude formation, these beliefs may also influence the degree of trust users place in the technology. When a system is perceived as useful and user-friendly, users are more likely to trust it in performing complex and high-stakes tasks such as clinical decision-making (9, 20).

- **H3:** TAM has a statistically significant effect on trust.
  - **H3a:** Perceived usefulness has a statistically significant effect on trust.
  - **H3b:** Perceived ease of use has a statistically significant effect on trust.

According to TAM, perceived usefulness and ease of use are direct predictors of behavioural intention. Numerous studies in healthcare IT adoption have confirmed that when clinicians believe a technology improves performance and is easy to use, they are more inclined to adopt it (16, 17, 26);

- **H4:** TAM has a statistically significant effect on behavioral intention.
  - **H4a:** Perceived usefulness has a statistically significant effect on behavioral intention.
  - **H4b:** Perceived ease of use has a statistically significant effect on behavioral intention.

Trust is a particularly salient factor in AI adoption due to the complexity, opacity, and high stakes associated with clinical applications. Physicians' trust in AI systems is expected to have a direct positive influence on their intention to adopt such technologies;

- **H5:** Trust has a statistically significant effect on behavioral intention.

#### 3.2.2 Mediating effect hypotheses

The following hypotheses examine whether trust mediates the relationship between other factors and behavioral intention:

Beyond direct effects, trust may act as a key mediating variable that transmits the effects of TPB components onto behavioural intention. For example, even if a physician perceives AI positively or feels normative pressure, actual intention may depend on the level of trust established;

In the healthcare AI, the mediating role of trust is critical due to the “black-box” nature of many algorithms. Studies have shown that even when an AI tool is technically efficient, clinicians' intention to adopt is often halted by a “trust gap” regarding the explainability of the AI's decisions. Therefore, trust serves as a necessary bridge, translating perceptions of usefulness and social influence into a concrete intention to integrate these tools into patient care (19, 23).

- **H6:** Trust mediates the relationship between TPB and behavioral intention.
  - **H6a:** Trust mediates the relationship between perceived behavioral control and behavioral intention.
  - **H6b:** Trust mediates the relationship between subjective norms and behavioral intention.
  - **H6c:** Trust mediates the relationship between attitude and behavioral intention.

Similarly, trust may mediate the relationship between TAM constructs and behavioural intention. Even when a system is perceived as useful and easy to use, physicians may only intend to use it if they also trust its reliability and relevance in clinical practice;

- **H7:** Trust mediates the relationship between the TAM and behavioral intention.
  - **H7a:** Trust mediates the relationship between perceived usefulness and behavioral intention.
  - **H7b:** Trust mediates the relationship between perceived ease of use and behavioral intention.

### 3.3 Research population

This study examines the acceptance and trust of AI technologies among physicians providing healthcare services in Türkiye. Therefore, the research population consists of physicians practicing in Türkiye. According to the 2024 data from the Ministry of Health, the total number of physicians in Türkiye was approximately 205,000 as of 2023 (27).

### 3.4 Statistical method

In this study, Structural Equation Modeling (SEM) was employed to analyze the relationships between dependent, independent, and mediating variables. Partial Least Squares Structural Equation Modeling (PLS-SEM) was preferred for this research, as it is well-suited for small data sets and effectively estimates complex relationships (28–31).

For data analysis, SmartPLS software was used (32). Additionally, Higher-Order Component (HOC) modeling and the Repeated Indicators Approach (RIA) were applied to construct higher-order constructs. These methods simplify complex structures, making them more manageable while enhancing the theoretical depth of the model (33).

### 3.5 Sample size

An *a priori* power analysis was conducted using G*Power software (34, 35) to determine the minimum required sample size for hypothesis testing. Based on a medium effect size (*f*^2^ = 0.15), a significance level of α = 0.05, and a desired statistical power of 1−β = 0.95, the analysis indicated that a minimum sample size of *N* = 153 was sufficient for PLS-SEM. Accordingly, the achieved sample size (*N* = 414) exceeded the minimum recommended threshold, ensuring adequate statistical power and robustness of the estimated model parameters.

A convenience sampling method was employed due to the exploratory nature of the study and the practical challenges of reaching physicians across multiple institutions nationwide. Online recruitment via professional networks and email enabled rapid access to a diverse physician population. This sampling approach is widely applied in healthcare technology acceptance research when probability-based sampling is not feasible. Therefore, sample adequacy was assessed based on established PLS-SEM power and minimum-sample recommendations rather than population-level representativeness (28, 36).

### 3.6 Data collection method

A structured survey was developed based on the proposed theoretical model. To enhance the reliability of the measurements, constructs were adapted from previously validated scales in prior research (5, 13, 17, 18, 37–39). The measurement instrument was reviewed by experts, and a pilot survey was conducted to assess its validity and reliability. Following expert review and pilot testing, minor wording refinements were made to improve clarity and ensure alignment with clinical terminology.

During measurement model assessment, two items (Subjective Norm 3 and Perceived Ease of Use 3) were removed due to low indicator loadings. This decision was not based solely on statistical thresholds. Conceptually, Subjective Norm 3 reflects organisational endorsement rather than perceived social pressure from salient referents, which constitutes the core definition of subjective norm in TPB. Perceived Ease of Use 3 partially overlaps with cognitive effort and perceived complexity constructs. Therefore, their removal was intended to preserve construct clarity and theoretical consistency while maintaining measurement validity.The final list of constructs used in this study, along with corresponding items and sources, is presented in Supplementary Table S1.

A quantitative research design was employed, and data were collected through a survey method. The questionnaire was designed using Google Forms and disseminated to the target population via social media and email following approval from the Ethics Committee. Physicians' responses were collected in an electronic database. A total of 414 physicians participated in the study between June 2023 and September 2023, and all responses were complete and included in the final analysis.

The survey consisted of two sections:

- Demographic and professional information: Participants provided details about their background and medical practice.
- Constructs related to TPB, TAM, and trust: Questions covered subjective norms, perceived behavioral control, perceived ease of use, perceived usefulness, attitude toward behavior, trust, and behavioral intention.

In this section, participants were asked to evaluate statements using a 5-point Likert scale, ranging from 1 = Strongly Disagree to 5 = Strongly Agree. The survey included 38 questions and was administered to 414 physicians working in various healthcare institutions in Türkiye.

### 3.7 Analysis process

The PLS-SEM analysis process in this study was conducted following the steps proposed by Hair et al. (28). These steps included: first, establishing reflective measurement relationships in the research model, followed by constructing the structural model. The model was then analyzed using the Hierarchical Component Model (HCM).

## 4 Results

A total of 414 physicians from 38 cities participated in the online survey, which was announced nationwide to physicians. Among the participants, 120 physicians (29%) stated that they use AI-based technologies in their institutions, while a total of 294 physicians (71%) reported that AI-based technologies are not used in their institutions. The demographic characteristics of the participants are presented in Table 1.

**Table 1:** Demographic Characteristics of the Participants.

Category	Data
Gender	
Female	174 (42%)
Male	240 (58%)
Age (year)	
≤25	4 (1%)
25–35	142 (34.3%)
36–45	107 (25.8%)
46–55	118 (28.5%)
≥56	43 (10.4%)
Title	
Fellow	99 (23.9%)
General Practitioner	19 (4.6%)
Specialist	132 (31.9%)
Assistant Professor	19 (4.6%)
Associate Professor	55 (13.3%)
Professor	90 (21.7%)
Institution	
Public	326 (78.7%)
Private	88 (21.3%)

### 4.1 Validity and reliability analyses of the research model

Before proceeding with hypothesis testing in the model, validity and reliability analyses were conducted within the scope of PLS-SEM. These analyses included an examination of internal consistency [Cronbach's alpha, composite reliability (CR)], convergent validity [indicator reliability, average variance extracted (AVE)], and discriminant validity [Fornell & Larcker criterion, Heterotrait-Monotrait ratio (HTMT)].

In evaluating internal consistency, Cronbach's alpha (α) and composite reliability (CR) were assessed against recommended thresholds (α ≥ 0.70 and CR ≥ 0.60) (33). Items Subjective Norm 3 (top management support) and Perceived Ease of Use 3 (mental effort) were removed from the final analysis as their factor loadings fell below the recommended thresholds. The removal of Subjective Norm 3 reflects that for physicians, professional autonomy and peer influence (Subjective Norm 1, 2 and 4) are more decisive factors in technology adoption than institutional hierarchy (Supplementary Table S1). Despite these removals, the Subjective Norm construct maintained strong internal consistency with a Composite Reliability (CR) of 0.801, well above the 0.70 threshold, ensuring the content validity and reliability of the scale were preserved (Table 2).

**Table 2:** Internal consistency assessment of the research model.

Sub-dimension	Cronbach's alpha (α)	Composite reliability (CR)
Perceived Behavioral Control	0.793	0.867
Subjective Norm	0.625	0.801
Attitude Toward Behavior	0.927	0.945
Perceived Usefulness	0.919	0.943
Perceived Ease of Use	0.791	0.878
Trust	0.804	0.871
Behavioral Intention	0.899	0.925

Following PLS-SEM guidelines for exploratory research, constructs with acceptable composite reliability were retained (28). Overall, composite reliability values met the accepted threshold for all sub-dimensions included in the study (Table 2).

To ensure convergent validity, the analysis of AVE values was conducted in the subsequent phase. Generally, AVE values are expected to be above 0.50 (28). The AVE values for the variables in the model are as follows: for the TPB factors, perceived behavioral control (0.621), subjective norm (0.577), and attitude toward behavior (0.775); for the TAM factors, perceived usefulness (0.806) and perceived ease of use (0.707); for the trust scale (0.630) and the behavioral intention scale (0.713) (Supplementary Table S4). It was determined that the AVE values are above 0.50 and that All indicator loadings exceeded the recommended threshold of 0.70 and ranged between 0.71 and 0.92 (28). Accordingly, the research model provides evidence of convergent validity.

To assess potential common method bias (CMB) in the PLS-SEM analysis, the study employed the Full Collinearity Assessment Approach recommended by Kock (40). This method evaluates both inner and outer variance inflation factor (VIF) values, using 3.3 as a conservative threshold for acceptable collinearity. Although this threshold is stringent, all VIF values in the model remained below the widely accepted limit of 5, indicating no critical multicollinearity among predictor constructs (28). Accordingly, CMB is unlikely to pose a threat to the validity of the results. The relevant VIF values are reported in Supplementary Table S4 (Supplementary Material). In addition, procedural remedies were implemented during data collection, including assurances of participant anonymity and confidentiality, to further reduce the risk of method bias.

For discriminant validity, cross-loadings, the Fornell-Larcker criterion, and HTMT 85/90 values were examined. The factor loading being greater than the cross-loadings is an indication of discriminant validity (28). It was observed that the loadings of all factors are greater than the cross-loadings (Supplementary Table S5).

Subsequently, the Fornell-Larcker table was examined (38). For this purpose, discriminant validity among the factors was assessed by comparing the square root of the AVE for each factor with the correlations between that factor and all other factors (28, 41). The results in the Fornell-Larcker table were obtained using the square root of the AVE values. It was observed that the obtained values were greater than all other correlation values when compared (Table 3).

**Table 3:** Measurement model discriminant validity: fornell-larcker table.

Fornell ve Larcker	PBC	SN	ATB	PU	PEU	T	BI
PBC	**0,788**						
SN	0,196	**0,760**					
ATB	0,318	0,378	**0,880**				
PU	0,309	0,458	0,691	**0,898**			
PEU	0,521	0,237	0,422	0,381	**0,841**		
T	0,368	0,418	0,399	0,446	0,473	**0,793**	
BI	0,298	0,354	0,786	0,709	0,431	0,478	**0,844**

ATB, attitude toward behavior; BI, behavioral intention; PBC, perceived behavioral control; PEU, perceived ease of use; PU, perceived usefulness; SN, subjective norm; T, trust.

Finally, the HTMT table was evaluated (Table 4) (33). It was found that all obtained values were less than 0.85, indicating that the research model provides evidence of discriminant validity.

**Table 4:** Measurement model discriminant validity: HTMT 85/90.

HTMT 85/90	PBC	SN	ATB	PU	PEU	T	BI
PBC							
SN	0,291						
ATB	0,371	0,482					
PU	0,363	0,588	0,746				
PEU	0,664	0,338	0,493	0,445			
T	0,449	0,593	0,452	0,512	0,578		
BI	0,355	0,460	0,858	0,778	0,514	0,555	

ATB, attitude toward behavior; BI, behavioral intention; PBC, perceived behavioral control; PEU, perceived ease of use; PU, perceived usefulness; SN, subjective norm; T, trust.

In conclusion, all tests and analyses conducted have confirmed the validity and reliability of the research model.

### 4.2 Research model analyses

This section presents the findings of the structural model analyses, organised in accordance with the proposed research framework. We first report the direct effects of TPB and TAM constructs on trust and behavioural intention, followed by an examination of the mediating role of trust. Hypothesis testing results and mediation analysis are structured to reflect the theoretical model.

#### 4.2.1 Direct effects of TPB and TAM constructs

Table 5 summarises the direct relationships between the constructs. Among the TPB constructs, perceived behavioural control (*β* = 0.111, *t* = 2.058, *p* = 0.040) and subjective norm (*β* = 0.237, *t* = 4.450, *p* < 0.001) had significant positive effects on trust, whereas attitude toward behaviour did not (*β* = 0.038, *t* = 0.630, *p* = 0.529). For behavioural intention, only attitude toward behaviour showed a significant direct effect (*β* = 0.537, *t* = 11.495, *p* < 0.001).

**Table 5:** Direct effects of TAM, TPB, and trust variables on behavioral intention.

Hypothesis	Tested relationship	*β*	t	*p*	CI		Result
					LB (2.5%)	UB (97.5%)	
H1a	Perceived Behavioral Control → Trust	0.111	2.058	0.040	0.007	0.219	Accepted
H1b	Subjective Norm → Trust	0.237	4.450	0.000	0.135	0.345	Accepted
H1c	Attitude Toward Behavior → Trust	0.038	0.630	0.529	−0.082	0.159	Rejected
H2a	Perceived Behavioral Control → Behavioral Intention	−0.033	0.883	0.377	−0.106	0.041	Rejected
H2b	Subjective Norm → Behavioral Intention	−0.045	1.278	0.201	−0.115	0.024	Rejected
H2c	Attitude Toward Behavior → Behavioral Intention	0.537	11.495	0.000	0.444	0.625	Accepted
H3a	Perceived Usefulness → Trust	0.171	2.950	0.003	0.055	0.282	Accepted
H3b	Perceived Ease of Use → Trust	0.277	5.170	0.000	0.172	0.383	Accepted
H4a	Perceived Usefulness → Behavioral Intention	0.284	6.373	0.001	0.196	0.372	Accepted
H4b	Perceived Ease of Use → Behavioral Intention	0.058	1.391	0.164	−0.023	0.140	Rejected
H5	Trust → Behavioral Intention	0.141	3.748	0.000	0.068	0.215	Accepted

CI, confidence interval; LB, lower bound; UB, upper bound., t statistics ≥ ± 1.96, *p* statistics ≥ 0.05, significance is established when the 95% bias-corrected and accelerated (BCa) CI does not inclode zero.

Within the TAM framework, both perceived usefulness (*β* = 0.171, *t* = 2.950, *p* = 0.003) and perceived ease of use (*β* = 0.277, *t* = 5.170, *p* < 0.001) were significantly associated with trust. In terms of behavioural intention, perceived usefulness had a significant direct effect (*β* = 0.284, *t* = 6.373, *p* = 0.001), while perceived ease of use did not (*β* = 0.058, *t* = 1.391, *p* = 0.164). Lastly, trust itself had a positive and statistically significant effect on behavioural intention (*β* = 0.141, *t* = 3.748, *p* < 0.001).

#### 4.2.2 Mediating role of trust

To evaluate the mediating role of trust, a series of indirect effect analyses were conducted. As shown in Table 6, trust was found to mediate the relationship between subjective norm and behavioural intention (*β* = 0.033, *t* = 2.864, *p* = 0.004), as well as between perceived usefulness and behavioural intention (*β* = 0.024, *t* = 2.357, *p* = 0.018), and perceived ease of use and behavioural intention (*β* = 0.039, *t* = 3.107, *p* = 0.002).

**Table 6:** Mediation Analysis Results.

Hypothesis	Tested relationship	*β*	t	*p*	CI		Result
					LB (2.5%)	UB (97.5%)	
H6a	Perceived Behavioral Control → Trust → Behavioral Intention	0.016	1.653	0.098	0.001	0.037	Rejected
H6b	Subjective Norm → Trust → Behavioral Intention	0.033	2.864	0.004	0.013	0.059	Accepted
H6c	Attitude Toward Behavior → Trust → Behavioral Intention	0.005	0.585	0.559	−0.012	0.026	Rejected
H7a	Perceived sefulness → Trust → Behavioral Intention	0.024	2.357	0.018	0.007	0.047	Accepted
H7b	Perceived Ease of Use → Trust → Behavioral Intention	0.039	3.107	0.002	0.017	0.066	Accepted

CI, confidence interval; LB, lower bound; UB, upper bound. t statistics ≥ ± 1.96, *p* statistics ≥ 0.05, Significance is established when the 95% bias-corrected and accelerated (BCa) CI does not inclode zero.

However, the mediating role of trust was not supported for perceived behavioural control (*β* = 0.016, *t* = 1.653, *p* = 0.098) or for attitude toward behaviour (*β* = 0.005, *t* = 0.585, *p* = 0.559). These findings suggest that trust functions as a selective mediator, operating primarily through constructs that reflect external ease and social influence, rather than internal volition or pre-formed attitudes.

### 4.3 Validation of higher-order structures

This study employed Higher Order Component and Second Order Construct methods to model abstract constructs such as TPB and TAM. Using the Repeated Indicator Approach, higher-order constructs were derived from their respective first-order constructs to enable a more comprehensive analysis of physician behaviour.

#### 4.3.1 Validity and reliability

Each higher-order construct was tested for internal consistency and convergent validity. Results for Cronbach's alpha (*α*), CR, and AVE were within acceptable thresholds: TPB (α = 0.560, CR = 0.773, AVE = 0.533), TAM (α = 0.552, CR = 0.817, AVE = 0.690), Trust (α = 0.804, CR = 0.871, AVE = 0.629), and Behavioral Intention (α = 0.899, CR = 0.925, AVE = 0.713). These metrics support both the reliability and convergent validity of the measurement model.

#### 4.3.2 Discriminant validity

The Fornell-Larcker criterion confirmed discriminant validity, as the square root of AVE for each construct exceeded its correlations with other constructs. This suggests that constructs are empirically distinct and that validity established at the first-order level is retained at the higher-order level.

### 4.4 Summary of results

#### 4.4.1 Summary of hypothesis test results

Table 7 presents the structural model results. TPB significantly influenced both trust and behavioural intention, while TAM had a significant effect on behavioural intention but not on trust. Trust showed a significant effect on behavioural intention (Table 8).

**Table 7:** Structural model hypothesis testing results.

Hypothesis	Path	*β*	*t*-value	*p*-value	Result
H1	TPB → Trust	0.498	3.863	<0.001	Supported
H2	TPB → Behavioral Intention	0.599	4.800	<0.001	Supported
H3	TAM → Trust	0.186	1.404	0.160	Not Supported
H4	TAM → Behavioral Intention	0.396	2.975	0.003	Supported
H5	Trust → Behavioral Intention	0.193	2.179	0.029	Supported

**Table 8:** Mediation analysis results.

Hypothesis	Indirect path	*β*	*p*-value	Result
H6	TPB → Trust → Behavioral Intention	0.096	0.040	Supported (Complementary Mediation)
H7	TAM → Trust → Behavioral Intention	−0.036	0.281	Not Supported

#### 4.4.2 Summary of mediation analysis

The mediation analysis revealed that trust significantly mediated the relationship between TPB and behavioral intention. Since both the direct and indirect effects are in the same positive direction, this indicates a “complementary mediation” effect. However, the mediating role of trust in the relationship between TAM and behavioral intention was not statistically supported.

## 5 Discussion

The increasing use of AI technologies in the healthcare sector has raised concerns, reluctance, and doubts among healthcare professionals regarding their applicability, potentially creating various challenges in integrating AI into the healthcare system. This study examines the factors that determine physicians' acceptance and intention to use AI technologies in clinical practice. Accordingly, the study integrates TPB and TAM frameworks. Additionally, following the guidance of previous studies, the role of trust is extensively analyzed as both an independent and mediating variable.

The findings of this study confirm that TPB, TAM, and trust have a significant impact on behavioral intention toward AI technology adoption (H2, H4, H5). These results are consistent with previous research (7, 9, 12, 13, 26, 39, 41–45). Furthermore, it was found that TPB has a stronger effect on behavioral intention compared to TAM and trust (β = 0.599 vs. 0.396 and 0.193). Similarly, Choudhury et al. reported in their study that TPB has a stronger predictive power than TAM (9). Taylor and Todd, in their comparative analysis of TAM and TPB for understanding the tendency to use information technologies, demonstrated that TPB provides a more comprehensive explanation of behavioral intention (18). On the other hand, in a study analyzing 125 research papers on technology acceptance in healthcare services, AlQudah et al. identified behavioral intention as the most commonly used assessment factor (8). Studies suggest that TAM and its subdimensions play a leading role in healthcare services, while TPB and its components serve as significant explanatory variables (8, 26, 41). Moreover, factors such as trust and anxiety have also been confirmed to influence technology adoption in healthcare services. This study further demonstrates that TPB, TAM, and trust have a significant impact on behavioral intention.

This study confirms that the core components of TAM, namely perceived ease of use and perceived usefulness, have a significant impact on behavioral intention. The findings indicate that users’ intention to adopt a technology is directly related to the effort required to use it and the expected benefits. The literature highlights perceived usefulness and ease of use as key determinants of technology acceptance (6, 16, 46). These results emphasize the importance of making AI technologies user-friendly and integrating motivational elements to support the adoption process.

Studies suggest that TAM and its subdimensions—particularly perceived usefulness and perceived ease of use—often emerge as primary determinants of technology adoption in healthcare, while TPB and its components provide significant explanatory power for behavioural intention (8, 25, 26, 47). On the other hand, when examining the effects of TPB subdimensions—perceived behavioral control, subjective norm, and attitude—on behavioral intention, only attitude was found to have a significant effect. This finding aligns with the study by Rahman et al. (46), while research by Zhou and Hsieh suggests that subjective norm and perceived behavioral control also play a role (15, 47). Similarly, a study conducted by Hadadgar on healthcare professionals have shown that attitude and perceived behavioral control are key determinants of technology adoption intention (11). In a study conducted in Germany, Perlich et al. identified attitude as the strongest predictor of technology adoption intention (48). The findings underscore the critical role of attitude in physicians' adoption of AI technologies, highlighting the need for attitude development strategies to ensure the successful integration of AI applications in healthcare services.

This study found that perceived behavioral control and subjective norm did not have a significant impact. Although these results contradict the original TPB model and some previous studies (8, 9), they are consistent with the findings of Sun and Zhang (49) and Aljarboa and Miah (50). This suggests that technology providers and healthcare institutions should develop strategies to enhance AI technology acceptance.

To better understand the role of trust in AI adoption, its antecedents were examined. The findings reveal that subjective norm, perceived behavioral control, perceived usefulness, and perceived ease of use have significant effects on trust. Users' social influences, their perceived competence, and their evaluations of the ease or benefits of a technology are key factors in trust formation. Fan et al. defined the antecedents of trust in technology adoption as individual experiences, perceptions of technology, and the institutional environment (20). Hackman and Knowlden highlighted that individuals tend to conform to the opinions of those they consider important within their social environment, demonstrating that subjective norms play a direct role in trust formation (22). Similarly, Li et al. emphasized the impact of social influence on trust, indicating a direct relationship between subjective norms and trust (51).

Particularly, individuals with limited knowledge or experience tend to develop trust in technology based on the opinions of people they perceive as reliable (52). This study supports the influence of subjective norms on trust and demonstrates that physicians take into account the attitudes of their colleagues and institutions toward AI technologies. Additionally, physicians' beliefs regarding the clinical benefits of these technologies, their potential to reduce workload, and their ease of use reinforce their sense of trust. A user-friendly design can accelerate adoption and facilitate clinical integration. The study specifically found that perceived ease of use had the strongest effect on trust compared to other variables (β = 0.277). This could be explained by the fact that easily usable technologies leave a positive impression during physicians' initial experiences and increase long-term adoption.

Healthcare professionals may have a higher sensitivity to risk compared to other users adopting AI technologies. Therefore, the primary concern in the acceptance of a new medical technology is not merely enhancing job performance but rather its potential impact on patients' lives and health. Trust is a fundamental factor that determines an individual's willingness to take risks; when perceived risks are high, individuals become more inclined to adopt new technologies. Previous studies have demonstrated that users' perceptions of benefit and ease of use have a direct impact on their trust in technology (9, 20, 21, 53–55). From the perspective of healthcare professionals, it can be anticipated that a higher level of expected benefit would lead to stronger trust in AI technologies. In this context, ease of use and expected benefit play a critical role in trust formation regarding AI technologies, whereas the direct influence of individuals' attitudes toward technology remains relatively limited. The findings suggest that strategies aimed at enhancing trust for the successful integration of AI technologies in healthcare services should be shaped around improving ease of use and strengthening benefit expectations. Establishing trust throughout the development, deployment, and usage processes of AI technologies is a critical requirement for realizing their full potential in the healthcare sector (55–57). Accordingly, trust is considered a fundamental variable in studies examining the adoption of AI technologies (49, 50). The results confirm that trust is closely related to the acceptance and intention to use AI technologies. This study highlights the necessity of expanding TAM and TPB by incorporating the trust factor to provide a more comprehensive framework for AI technology adoption. Trust serves as a central component in interactions between humans and AI technologies, and incorrect levels of trust emerge as a significant barrier to technology adoption (7).

The results of the mediation analysis reveal that trust plays a significant mediating role in the relationship between perceived behavioral control, subjective norm, perceived usefulness, perceived ease of use, and behavioral intention. This finding aligns with Fan et al.'s study on the adoption of AI-based diagnostic systems by healthcare professionals (20). Similarly, Choudhury et al. also identified trust as a mediating variable in the relationship between effort expectancy, performance expectancy, and behavioral intention (9). Given that effort expectancy corresponds to perceived ease of use and performance expectancy to perceived usefulness, a consistency between this study and previous findings can be observed.

On the other hand, it was found that trust eliminates the effect of attitude on behavioral intention. This indicates that in scenarios where trust is high, individuals' attitudes—whether positive or negative—do not significantly influence their behavioral intentions. In other words, trust may be a stronger determinant in shaping individuals' behavioral intentions than attitude. Accordingly, perceived behavioral control, subjective norm, perceived usefulness, and perceived ease of use are key factors influencing behavioral intention through trust.

According to Hair et al.'s classification of mediation analysis, complementary mediation was observed for perceived usefulness, whereas only indirect mediation was found for perceived behavioral control, subjective norm, and perceived ease of use (28). This means that perceived usefulness can directly influence behavioral intention while also strengthening this effect indirectly through trust. In contrast, other factors influence behavioral intention not directly, but only through mediating variables such as trust or attitude. Additionally, it was observed that trust mediates the relationship between TPB and behavioral intention, but it does not have the same effect in the relationship between TAM and behavioral intention. In this context, optimizing the levels of trust and concern is a critical requirement for encouraging healthcare professionals to adopt AI technologies. Specifically, increasing awareness among healthcare professionals regarding their roles and responsibilities in AI technologies may strengthen their sense of trust. In conclusion, trust is a decisive factor in the adoption of AI technologies, and strategies should be developed to enhance users' trust to ensure the successful integration of these technologies.

The findings confirm the pivotal role of trust as a bridge between TPB components and behavioral intention. The significant indirect effect (*ß* = 0.096, *p* = 0.040) suggests that social influences and perceived control are translated into actual adoption intent through the establishment of trust in AI systems. Conversely, the lack of a significant mediating path for TAM constructs (H7) implies that when AI is perceived as highly useful and easy to use, physicians may form an intention to adopt it directly, potentially bypassing the trust formation stage. This underscores that while trust is a key driver for socially-driven adoption, functional excellence in AI design can independently stimulate physician acceptance. Similar findings have been reported in earlier technology acceptance studies where trust did not independently predict adoption in low-experience environments (48, 49).

Finally, cultural or institutional factors might play a role. In hierarchical healthcare systems, decisions about technology use are often externally mandated, potentially reducing the weight of individual trust in shaping intention. These nuances suggest that the role of trust in AI adoption may evolve with increased system maturity and clinical familiarity. Future studies should examine trust development over time and across different healthcare contexts.

### 5.1 Limitations

This study has several limitations. First, the sample was limited to physicians in Türkiye, which may affect the generalisability of the results. Second, the electronic questionnaire did not include a standardised definition or baseline explanation of AI technologies, which may have led to variability in how respondents interpreted the term. Third, the study did not explore the influence of demographic factors or clinical specialties, limiting deeper insights into contextual differences. A key limitation of this study is the absence of a standardised operational definition of “AI technologies” provided to participants. Given the broad and heterogeneous nature of AI applications in healthcare, and as the questionnaire was distributed electronically without additional clarification, respondents may have interpreted the term differently based on their own experiences. This variation could have affected the consistency and validity of the responses.

Based on the identified limitations, several directions for future research are proposed. First, to address the lack of conceptual clarity, future studies should provide participants with a standardised and clearly operationalised definition of AI technologies, ensuring consistent interpretation across diverse medical backgrounds. Second, given that this study captures a snapshot of the early adoption phase in Türkiye, longitudinal research designs are needed to examine how physicians’ trust and behavioural intention evolve as AI systems become more integrated into clinical workflows. Third, future studies should employ probability sampling or multi-country comparative designs to enhance the generalisability of findings across different healthcare systems and cultures. Furthermore, incorporating specific clinical specialties and demographic factors would allow for a more nuanced understanding of how acceptance varies across different fields of medicine. Finally, moving beyond quantitative models, qualitative or mixed-method approaches are recommended to explore the deep-seated psychological and ethical reasons behind physicians' trust formation and resistance toward AI technologies.

Also, trust in this study was primarily operationalised as competence-based trust in AI systems. Other dimensions of trust, such as benevolence and integrity beliefs, were not explicitly measured. Future studies should adopt multidimensional trust scales to examine how different trust dimensions jointly influence physicians' adoption of AI technologies.

### 5.2 Contributions

#### 5.2.1 Theoretical contributions

This study contributes to the theoretical understanding of technology acceptance in healthcare by integrating TAM and TPB into a unified framework. Unlike prior studies that typically apply these models in isolation, our research demonstrates the complementary strengths of both frameworks when used together. Specifically, TAM provides insight into system-related beliefs, while TPB captures social and behavioural determinants of adoption.

Furthermore, the introduction of trust as a mediating variable represents a meaningful theoretical extension. Trust has been underexplored in previous TAM/TPB-based models, particularly in the context of AI adoption in clinical decision-making. Our findings underscore the importance of trust in bridging perceptions of technology features with behavioural intentions, thereby enhancing the predictive power of existing models. Additionally, the study enriches the literature by examining AI acceptance in a middle-income country context (Türkiye), offering culturally specific insights that may inform future cross-national studies.

#### 5.2.2 Practical implications

The findings of this study offer several actionable insights for healthcare stakeholders aiming to promote AI adoption among physicians. First, the strong influence of trust on behavioural intention suggests that healthcare institutions should prioritise building trust through transparent communication, ethical governance, and evidence-based validation of AI systems. Initiatives such as clinical demonstrations, peer endorsement, and real-world case studies may improve trust and perceived safety.

Second, the significance of perceived behavioural control and subjective norms indicates that training programmes must extend beyond basic technical skills to include confidence-building and role modelling. Peer-led education and institutional support mechanisms can reinforce positive attitudes and alleviate uncertainty. Policymakers and hospital administrators should also develop formal guidelines and integrate AI competencies into continuous professional development plans.

Finally, given the model's empirical relevance in a middle-income healthcare setting, the findings provide a roadmap for similar health systems seeking to advance AI integration in a socially and culturally responsive manner.

### 5.3 Future research

Future studies should replicate and extend the model across diverse medical specialties, professions, and countries. Research could also test the influence of specific trust-building interventions and assess long-term adoption patterns. From a design perspective, developers should focus on increasing transparency and clinical relevance to enhance trust.

An alternative theoretical framework that could be considered in future studies is the UTAUT. UTAUT integrates key elements of TAM, TPB, and other acceptance models into a comprehensive framework and has been widely applied in technology adoption research (10). Future research may apply UTAUT-based models to compare their explanatory power with the integrated TAM–TPB–trust model used in the present study, thereby offering further insights into physicians' adoption of AI technologies.

## 6 Conclusion

This study offers important insights into the behavioural mechanisms underlying physicians' adoption of AI in clinical settings. It confirms that TPB and TAM are both valid frameworks in this domain, with TPB exhibiting a stronger predictive capacity. Trust emerged as a critical determinant, significantly mediating the impact of TPB components on behavioral intention, which highlights the necessity of building confidence in AI systems to foster physician adoption. The results suggest that integration strategies should prioritise ease of use, perceived usefulnesss, and institutional support to foster positive attitudes and trust among physicians.

## Data Availability

The raw data supporting the conclusions of this article will be made available by the authors, without undue reservation.

## References

[1] Roppelt JS Kanbach DK Kraus S. Artificial intelligence in healthcare institutions: a systematic literature review on influencing factors. Technol Soc. (2024) 76:102443. 10.1016/j.techsoc.2023.102443

[2] Khanijahani A Iezadi S Dudley S Goettler M Kroetsch P Wise J. Organizational, professional, and patient characteristics associated with artificial intelligence adoption in healthcare: a systematic review. Health Policy Technol. (2022) 11(1):100602. 10.1016/j.hlpt.2022.100602

[3] Goirand M Austin E Clay-Williams R. Implementing ethics in healthcare AI-based applications: a scoping review. Sci Eng Ethics. (2021) 27(5):61. 10.1007/s11948-021-00336-3 34480239

[4] Castagno S Khalifa M. Perceptions of artificial intelligence among healthcare staff: a qualitative survey study. Front Artif Intell. (2020) 3:578983. 10.3389/frai.2020.578983 33733219 PMC7861214

[5] Venkatesh V Davis FD. A theoretical extension of the technology acceptance model: four longitudinal field studies. Manage Sci. (2000) 46(2):186–204. 10.1287/mnsc.46.2.186.11926

[6] Taylor S Todd PA. Understanding information technology usage: a test of competing models. Inf Syst Res. (1995) 6(2):144–76. 10.1287/isre.6.2.144

[7] Wu I-L Chen J-L. An extension of trust and TAM model with TPB in the initial adoption of online tax: an empirical study. Int J Hum Comput Stud. (2005) 62(6):784–808. 10.1016/j.ijhcs.2005.03.003

[8] AlQudah AA Al-Emran M Shaalan K. Technology acceptance in healthcare: a systematic review. Appl Sci. (2021) 11(22):10537. 10.3390/app112210537

[9] Choudhury A Asan O Medow JE. Effect of risk, expectancy, and trust on clinicians’ intent to use an artificial intelligence system. Appl Ergon. (2022) 101:103708. 10.1016/j.apergo.2022.103708 35149301

[10] Venkatesh V Morris MG Davis GB ve Davis FD. User acceptance of information technology: toward A unifed vie. MIS Q. (2003) 27(3):425–78. 10.2307/30036540

[11] Ajzen I. The theory of planned behavior. Organ Behav Hum Decis Process. (1991) 50(2):179–211. 10.1016/0749-5978(91)90020-T

[12] Hadadgar A Changiz T Masiello I Dehghani Z Mirshahzadeh N Zary N. Applicability of the theory of planned behavior in explaining the general practitioners’ e-learning use in continuing medical education. BMC Med Educ. (2016) 16:215. 10.1186/s12909-016-0738-6 27549190 PMC4994161

[13] Beck L Ajzen I. Predicting dishonest actions using the theory of planned behavior. J Res Pers. (1991) 25(3):285–301. 10.1016/0092-6566(91)90021-H

[14] Ursavaş ÖF Yalçın Y Bakır E. The effect of subjective norms on preservice and in-service teachers’ behavioural intentions to use technology: a multigroup multimodel study. Br J Educ Technol. (2019) 50(5):2501–19. 10.1111/bjet.12834

[15] Zhou Y Thøgersen J Ruan Y Huang G. The moderating role of human values in planned behavior: the case of Chinese consumers’ intention to buy organic food. J Consum Mark. (2013) 30(4):335–44. 10.1108/JCM-02-2013-0482

[16] Davis FD. Perceived usefulness, perceived ease of use, and user acceptance of information technology. MIS Q. (1989) 13(3):319–40. 10.2307/249008

[17] Rho MJ Choi IY Lee J. Predictive factors of telemedicine service acceptance and behavioral intention of physicians. Int J Med Inf. (2014) 83(8):559–71. 10.1016/j.ijmedinf.2014.05.005 24961820

[18] Prakash AV Das S. Medical practitioners’ adoption of intelligent clinical diagnostic decision support systems: a mixed-methods study. Inf Manag. (2021) 58(7):103524. 10.1016/j.im.2021.103524

[19] Weber S Wyszynski M Godefroid M Plattfaut R Niehaves B. How do medical professionals make sense (or not) of AI? A social-media-based computational grounded theory study and an online survey. Comput Struct Biotechnol J. (2024) 24:146–59. 10.1016/j.csbj.2024.02.009 38434249 PMC10904922

[20] Fan W Liu J Zhu S Pardalos PM. Investigating the impacting factors for healthcare professionals to adopt artificial intelligence-based medical diagnosis support systems (AIMDSS). Ann Oper Res. (2020) 294:567–92. 10.1007/s10479-018-2818-y

[21] Li X Hess TJ Valacich JS. Why do we trust new technology? A study of initial trust formation with organizational information systems. J Strat Inf Syst. (2008) 17(1):39–71. 10.1016/j.jsis.2008.01.001

[22] Hackman CL Knowlden AP. Theory of reasoned action and theory of planned behavior-based dietary interventions in adolescents and young adults: a systematic review. Adolesc Health Med Ther. (2014) 5:101–14. 10.2147/AHMT.S56207 24966710 PMC4057331

[23] Zhou C Liu X Yu C Tao Y Shao Y. Trust in AI-augmented design: applying structural equation modeling to AI-augmented design acceptance. Heliyon. (2024) 10(1):e23305. 10.1016/j.heliyon.2023.e23305 38192792 PMC10771990

[24] Shevtsova D Ahmed A Boot IWA Sanges C Hudecek M Jacobs JJL Trust in and acceptance of artificial intelligence applications in medicine: mixed methods study. JMIR Hum Factors. (2024) 11:e47031. 10.2196/47031 38231544 PMC10831593

[25] Holden RJ Karsh BT. The technology acceptance model: its past and its future in health care. J Biomed Inform. (2010) 43(1):159–72. 10.1016/j.jbi.2009.07.002 19615467 PMC2814963

[26] King WR He J. A meta analysis of the technology acceptance model. Inf Manag. (2006) 43:740–55. 10.1016/j.im.2006.10.007

[27] Available online at: https://dosyasb.saglik.gov.tr/Eklenti/48055/0/siy2022eng050420241pdf.pdf

[28] Hair JF Jr Hult GTM Ringle CM Sarstedt M. A Primer on Partial Least Squares Structural Equation Modeling (PLS-SEM). 2nd ed. Thousand Oaks: Sage publications (2017).

[29] Hair JF Jr Hult GTM Ringle CM Sarstedt M Danks NP Ray S. Partial Least Squares Structural Equation Modeling (PLS-SEM) Using R: A Workbook. Cham: Springer Nature (2021). p. 197. Available online at: https://library.oapen.org/handle/20.500.12657/51463

[30] Hair JF ve Sarstedt M. Factors versus composites: guidelines for choosing the right structural equation modeling method. Proj Manag J. (2019) 50(6):619–24. 10.1177/8756972819882132

[31] Hair J Alamer A. Partial least squares structural equation modeling (PLS-SEM) in second language and education research: guidelines using an applied example. Res Methods Appl Linguist. (2022) 1(3):100027. 10.1016/j.rmal.2022.100027

[32] Ringle C. Wende S Becker JM. (2023). SmartPLS 4. Available online at: https://smartpls.com/faq/smartpls4/how-to-cite-smartpls

[33] Hair JF Sarstedt M Ringle CM Gudergan SP. Advanced Issues in Partial Least Squares Structural Equation Modeling (PLS-SEM). 2nd ed. Thousand Oaks: Sage (2024).

[34] Faul F Erdfelder E Lang A-G Buchner A. G*Power 3: a flexible statistical power analysis program for the social, behavioral, and biomedical sciences. Behav Res Methods. (2007) 39:175–91. 10.3758/bf03193146 17695343

[35] Faul F Erdfelder E Buchner A Lang A-G. Statistical power analyses using G*Power 3.1: tests for correlation and regression analyses. Behav Res Methods. (2009) 41:1149–60. 10.3758/BRM.41.4.1149 19897823

[36] Saunders M Lewis P Thornhill A. Research Methods for Business Students. London: Prentice Hall (2009). p. 2009. ISBN, 0273716867, 9780273716860.

[37] Ajzen I Fishbein M. Attitude-behavior relations: a theoretical analysis and review of empirical research. Psychol Bull. (1977) 84(5):888–918. 10.1037/0033-2909.84.5.888

[38] Fornell C Larcker DF. Evaluating structural equation models with unobservable variables and measurement error. J Mark Res. (1981) 18(1):39–50. 10.2307/3151312

[39] Choung H David P Ross A. Trust in AI and its role in the acceptance of AI technologies. Int J Hum Comput Interaction. (2023) 39(9):1727–39. 10.1080/10447318.2022.2050543

[40] Kock N. Common method bias in PLS-SEM: a full collinearity assessment approach. Int J e-Collab. (2015) 11(4):1–10. 10.4018/IJeC.2015100101

[41] Lee Y Kozar KA Larsen KR. The technology acceptance model: past, present, and future. Commun Ais. (2003) 12(1):50. 10.17705/1CAIS.01250

[42] Chow WS Chen Y. Intended belief and actual behavior in green computing in Hong Kong. J Comput Inf Syst. (2009) 50(2):136–41. 10.1080/08874417.2009.11645392

[43] Chang HH Wang IC. An investigation of user communication behaviour in computer mediated environments. Comput Hum Behav. (2008) 24:2336–56. 10.1016/j.chb.2008.01.001

[44] Cheung R Vogel D. Predicting user acceptance of collaborative technologies: an extension of the technology acceptance model for E-learning. Comput Educ. (2013) 63:160–75. 10.1016/j.compedu.2012.12.003

[45] Stevens AF Stetson P. Theory of trust and acceptance of artificial intelligence technology (TrAAIT): an instrument to assess clinician trust and acceptance of artificial intelligence. J Biomed Inform. (2023) 148:104550. 10.1016/j.jbi.2023.104550 37981107 PMC10815802

[46] Rahman MNA Seyal AH ve Tajuddin ST. Exploring whatsapp adoption among students using the theory of planned behavior. In: Seyal AH Rahman MNA, editors. Theory of Planned Behavior New Research. New York: Nova Science Publishers (2017). p. 181–99.

[47] Hsieh PJ. Healthcare professionals’ use of health clouds: integrating technology acceptance and status quo bias perspectives. Int J Med Inf. (2015) 84(7):512–23. 10.1016/j.ijmedinf.2015.03.004 25842155

[48] Perlich A Meinel C Zeis D. Evaluation of the technology acceptance of a collaborative documentation system for addiction therapists and clients. Stud Health Technol Inform. (2018) 247:695–9. 10.3233/978-1-61499-852-5-695 29678050

[49] Sun H Zhang P. The role of moderating factors in user technology acceptance. Int J Hum Comput Stud. (2006) 64:53–78. 10.1016/j.ijhcs.2005.04.013

[50] Aljarboa S Miah SJ. Acceptance of clinical decision support systems in Saudi healthcare organisations. Inf Dev. (2023) 39(1):86–106. 10.1177/02666669211025076

[51] Li X Hess TJ Valacich JS. Using attitude and social influence to develop an extended trust model for information systems. ACM SIGMIS Database DATABASE Adv Inf Syst. (2006) 37(2–3):108–24. 10.1145/1161345.1161359

[52] Ryu K ve Jang S. Intention to experience local cuisine in a travel destination: the modified theory of reasoned action. J Hosp Tour Res. (2006) 30(4):507–16. 10.1177/1096348006287163

[53] Gefen D Rose GM Warkentin M Pavlou PA. Cultural diversity and trust in IT adoption: a comparison of potential e-voters in the USA and South Africa. J Glob Inf Manag. (2005) 13(1):55–79. 10.4018/978-1-59904-939-7.ch216

[54] Tung FC Chang SC Chou CM. An extension of trust and TAM model with IDT in the adoption of the electronic logistics information system in HIS in the medical industry. Int J Med Inf. (2008) 77(5):324–35. 10.1016/j.ijmedinf.2007.06.006 17644029

[55] Gu Z Wei J. Empirical study on initial trust of wearable devices based on product characteristics. J Comput Inf Syst. (2020) 61(6):520–8. 10.1080/08874417.2020.1779150

[56] European Commission: Directorate-General for Communications Networks, Content and Technology, Ethics guidelines for trustworthy AI, Publications Office. (2019). Available online at: https://data.europa.eu/doi/10.2759/346720

[57] Simion M Kelp C. Trustworthy artificial intelligence. AJPH. (2023) 2:8. 10.1007/s44204-023-00063-5

