# Supplementary material for: How physicians embrace AI: insights from technology acceptance and trust theories
Source: Front Digit Health. 2026 Mar 13;8:1722087. doi: 10.3389/fdgth.2026.1722087 (PMC13021828; doi:10.3389/fdgth.2026.1722087)
Supplement: Supplementary file 1 [file Datasheet1.pdf]

Table 1. Variables and their references

Latent Variable	Observed Variable	Reference
**Subjective Norms**	SN1 I believe that my colleagues. whose opinions I value. support the use of AI-based technologies in practice.	5, 17,44
	SN2 I believe that the professional associations I am a member of. which operate in the healthcare field. support the use of AI-based technologies in practice.	
	SN3 I believe that the use of AI-based technologies is supported by my institution's senior management.	
	SN4 I believe that many physicians worldwide use AI-based technologies.	
**Perceived Behavioral Control**	PBC1 I have the necessary resources to use AI-based technologies.	6,55
	PBC2 I have the necessary knowledge to use AI-based technologies.	
	PBC3 Using AI-based technologies is entirely under my control.	
	PBC4 I have the necessary skills to use AI-based technologies.	
**Perceived Ease of Use**	PEU1 Using AI-based technologies is easy.	5
	PEU2 The use of AI-based technologies is clear and understandable.	
	PEU3 Using AI-based technologies does not require excessive mental effort.	
	PEU4 I find it easy to get AI-based technologies to perform the tasks I expect.	
**Perceived Usefulness**	PU1 Using AI-based technologies enhances my work performance.	5
	PU2 Using AI-based technologies increases my work efficiency.	
	PU3 Using AI-based technologies improves my work effectiveness.	
	PU4 I find AI-based technologies useful in my work.	
**Attitude Toward Behavior**	ATB1 I feel positive about AI-based technologies.	46
	ATB2 I think using AI-based technologies is enjoyable.	
	ATB3 I find the use of AI-based technologies necessary.	
	ATB4 Using AI-based technologies is a good idea.	
	ATB5 Using AI-based technologies is a smart way to get things done.	
**Trust**	T1 AI-based technologies function well.	46
	T2 AI-based technologies have the necessary features to complete essential tasks.	
	T3 AI-based technologies are competent in their specific fields.	
	T4 AI-based technologies are reliable.	
**Behavioral Intention**	BI1 I intend to use AI-based technologies in the future.	13
	BI2 I plan to use AI-based technologies frequently.	
	BI3 I predict that I will use AI-based technologies in the future.	
	BI4 I expect my use of AI-based technologies to continue in the future.	
	BI5 I intend to recommend the use of AI-based technologies to others.	

Attitude Toward Behavior (ATB). Behavioral Intention (BI). Perceived Behavioral Control (PBC). Perceived Ease of Use (PEU). Perceived Usefulness (PU). Subjective Norm (SN) and Trust (T).

Table 4. Internal Consistency Results for the Measurement Model

	β	α	rho_A	CR	AVE	VIF
**PBC**		0.793	0.795	0.867	0.621	
**PBC1**	0.835					2.066
**PBC2**	0.861					2.371
**PBC3**	0.741					1.446
**PBC4**	0.704					1.411
**SN**		0.625	0.659	0.801	0.577	
**SN1**	0.836					1.438
**SN2**	0.818					1.477
**SN3**	Since the factor loading is less than 0.50. it has been removed.					
**SN4**	0.603					1.102
**ATB**		0.927	0.928	0.945	0.775	
**ATB1**	0.905					3.705
**ATB2**	0.884					3.196
**ATB3**	0.883					3.444
**ATB4**	0.877					3.320
**ATB5**	0.850					2.442
**PU**		0.919	0.921	0.943	0.806	
**PU1**	0.925					4.184
**PU2**	0.934					4.624
**PU3**	0.863					2.546
**PU4**	0.867					2.472
**PEU**		0.791	0.791	0.878	0.707	
**PEU1**	0.873					2.354
**PEU2**	0.873					2.270
**PEU3**	Since the factor loading is less than 0.708. it has been removed.					
**PEU4**	0.772					1.335
**T**		0.804	0.816	0.871	0.630	
**T1**	0.841					1.856
**T2**	0.815					1.819
**T3**	0.740					1.546
**T4**	0.775					1.547

**BI**		0.899	0.901	0.925	0.713	
**BI1**	0.813					2.290
**BI2**	0.812					2.213
**BI3**	0.850					2.622
**BI4**	0.903					3.636
**BI5**	0.840					2.474

Attitude Toward Behavior (ATB). Behavioral Intention (BI). Perceived Behavioral Control (PBC). Perceived Ease of Use (PEU). Perceived Usefulness (PU). Subjective Norm (SN) and Trust (T).

Table 5. Discriminant Validity of the Measurement Model - Cross Loadings

Dual Cross Loadings	PBC	SN	ATB	PU	PEU	T	BI
**PBC1**	0.835	0.151	0.219	0.230	0.336	0.340	0.226
**PBC2**	0.861	0.158	0.260	0.247	0.422	0.247	0.240
**PBC3**	0.741	0.119	0.183	0.206	0.404	0.282	0.233
**PBC4**	0.704	0.189	0.341	0.289	0.485	0.278	0.239
**SN1**	0.142	0.836	0.381	0.439	0.207	0.336	0.353
**SN2**	0.110	0.818	0.261	0.367	0.147	0.341	0.235
**SN4**	0.212	0.603	0.194	0.205	0.188	0.273	0.197
**ATB1**	0.274	0.315	0.905	0.643	0.354	0.326	0.699
**ATB2**	0.327	0.324	0.884	0.628	0.404	0.374	0.716
**ATB3**	0.270	0.315	0.883	0.543	0.366	0.298	0.649
**ATB4**	0.230	0.316	0.877	0.573	0.350	0.318	0.679
**ATB5**	0.292	0.389	0.850	0.645	0.380	0.427	0.707
**PU1**	0.278	0.411	0.652	0.925	0.349	0.409	0.664
**PU2**	0.269	0.416	0.646	0.934	0.346	0.380	0.671
**PU3**	0.275	0.402	0.614	0.863	0.310	0.379	0.598
**PU4**	0.289	0.416	0.570	0.867	0.362	0.434	0.609
**PEU1**	0.496	0.179	0.368	0.303	0.873	0.338	0.364
**PEU2**	0.433	0.214	0.331	0.314	0.873	0.440	0.352
**PEU4**	0.388	0.200	0.366	0.340	0.772	0.405	0.369
**T1**	0.368	0.354	0.357	0.399	0.446	0.841	0.421
**T2**	0.307	0.311	0.315	0.362	0.413	0.815	0.350
**T3**	0.217	0.354	0.238	0.273	0.265	0.740	0.287
**T4**	0.254	0.315	0.339	0.365	0.351	0.775	0.440
**BI1**	0.294	0.258	0.616	0.580	0.389	0.406	0.813
**BI2**	0.271	0.239	0.621	0.550	0.432	0.351	0.812
**BI3**	0.228	0.359	0.678	0.630	0.319	0.416	0.850
**BI4**	0.274	0.315	0.730	0.639	0.358	0.385	0.903
**BI5**	0.194	0.316	0.667	0.590	0.330	0.457	0.840

Attitude Toward Behavior (ATB). Behavioral Intention (BI). Perceived Behavioral Control (PBC). Perceived Ease of Use (PEU). Perceived Usefulness (PU). Subjective Norm (SN) and Trust (T).
